# Guideline based knowledge and practice of physicians in the management of COPD in a low‐ to middle‐income country

**DOI:** 10.1111/crj.13468

**Published:** 2022-01-13

**Authors:** Suraj Ghimire, Anish Lamichhane, Anita Basnet, Samiksha Pandey, Nahakul Poudel, Bhushan Shrestha, Santosh Pathak, Gaurav Mahato, Ram Kumar Shrestha

**Affiliations:** ^1^ School of Medicine and Public Health University of Newcastle Callaghan New South Wales Australia; ^2^ Department of Pediatrics Lincoln Medical and Mental Health Centre New York New York USA; ^3^ Nepal Health Sector Support Program Lalitpur Nepal; ^4^ Department of Internal Medicine Beaumont Health Royal Oak Michigan USA; ^5^ Maharajgunj Medical Campus Tribhuvan University Kathmandu Nepal; ^6^ Janaki Medical College Tribhuvan University Janakpur Nepal; ^7^ G. P. Koirala National Centre for Respiratory Disease Shuklagandaki Nepal

**Keywords:** COPD, guideline, knowledge, Nepal, physicians, practice

## Abstract

**Background:**

Chronic obstructive pulmonary disease (COPD) is the third leading cause of death, with 80% of the total death occurring in low‐ to middle‐income countries (LMICs). Nepal is one of the LMIC; COPD is a highly prevalent and significant public health issue often underdiagnosed. Medical physicians' good knowledge and practice to diagnose and treat COPD can help reduce the disease burden.

**Objectives:**

To determine the level of knowledge, practice and factors influencing the practice of physicians regarding COPD management based on GOLD guidelines.

**Design:**

A cross‐sectional descriptive study using a structured questionnaire was conducted among medical physicians working in Bagmati and Gandaki province of Nepal. Out of total scores, physicians knowledge and practice were graded according to Bloom's original cut‐off point for good (≥80%), satisfactory (60%–78%) and poor (<60%).

**Result:**

A total of 152 medical physicians participated in this study. Out of the possible total score 20, the mean score on knowledge was 17.8 ± 2.4, and out of possible total score eight, the mean score on practice was 5.3 ± 1.3. The correlation test between total knowledge and practice scores showed *r* = 0.18 and *p* value <0.02. The most selected factors hindering the appropriate management of COPD was lack of patient follow up and lack of professional training in COPD. Other factors included patient unwillingness to discuss smoking quit plan, lack of screening tool, unavailability of spirometry and physician unawareness of available medicine to treat COPD.

**Conclusion:**

Despite physicians having good knowledge in COPD, the practice in COPD management is below guideline‐recommended. There is a significant, very low positive correlation between total knowledge score and practice score. Proper COPD training to physicians, disease awareness among patients, easy availability of diagnostic equipment and medication can help improve physicians' practice and appropriately manage COPD patients.

## 1 INTRODUCTION

Chronic obstructive pulmonary disease (COPD) is defined as a common, preventable and treatable disease characterised by persistent respiratory symptoms and airflow limitation due to airway or alveolar abnormalities usually caused by significant exposure to noxious particles or gases.^1^ It is the 4th leading cause of death globally and is projected to be the third leading cause by 2020.^2^ There is a significant variation in COPD prevalence worldwide, with 10%–95% underdiagnosis and 5%–60% overdiagnosis due to differences in the definition of diagnosis used and the unavailability of spirometry, especially in rural areas of low and middle‐income countries (LMICs).^3^ According to the National Burden of Disease 2017 report in Nepal, non‐communicable disease (NCD) accounts for significant deaths and 1 in 10 deaths (10% of total deaths) is attributed to COPD.^4^

Smoking is one of the most critical risk factors for COPD.^5^, ^6^ Other risk factors include a family history of COPD, second‐hand smoking, exposure to cooking or home heating fuels, and occupational specks of dust/chemicals.^7^ COPD should be considered in any patient with dyspnoea, chronic cough or sputum production, and a history of recurrent lower respiratory tract infections with or without a history of exposure to harmful particles or gases.^8^ The US National Heart, Lung, and Blood Institute and the World Health Organization established the Global Initiative for Chronic Obstructive Lung Disease (GOLD) to prepare a standard guideline for COPD management. The primary purpose of this project was to create and disseminate guidelines that would help prevent COPD and establish a standard of care for treating patients with COPD based on the most current medical evidence. As per GOLD, spirometry is needed to diagnose COPD with the post‐bronchodilator value of FEV1/FVC < 0.70, confirming the presence of persistent airflow limitation.^9^ Despite much advancement in COPD treatment, studies suggest either underdiagnosis or overdiagnosis of the disease. A study indicates that 80% of COPD cases confirmed by spirometry were underdiagnosed.^10^ Nepal being one of the resource‐poor countries, it is evident that COPD is a highly prevalent and significant public health issue that is often underdiagnosed.^11^ It is related to various factors like lack of spirometry facility, lack of awareness of various risk factors of COPD, such as exposure to biomass fuel, a low level of education in patients, and lack of disease awareness among healthcare providers.^12^ Proper knowledge of the disease and availability of adequate resources is required to implement the guideline and help doctors diagnose and provide best practices for COPD patients. Good practice based on proper guidelines can help diagnose the cases early and decrease COPD‐related morbidity and mortality.

No study is available in Nepal to look into the knowledge level of a medical physician on COPD, their practice, and barriers to guideline‐based management. Thus, we aim to study the knowledge among medical physicians from Gandaki and Bagmati province of Nepal on COPD as per GOLD guidelines, their current practice, and study factors influencing the proper management of COPD patients. Our findings can help the concerned authority plan and effectively mobilise medical physicians to decrease the morbidity and mortality secondary to COPD.

## 2 MATERIALS AND METHODS

### 2.1 Study design

We carried out a descriptive cross‐sectional study among medical physicians working in all levels of health care delivery in the Bagmati and Gandaki provinces of Nepal.

### 2.2 Study settings

Nepal is divided into seven provinces; we did the study in two provinces, that is, Bagmati and Gandaki. These were selected based on the highest number of COPD diagnosed in the last 5 years.^13^ We categorised health facilities into primary health facilities, secondary health facilities, tertiary health facilities and private practice. Primary level health facilities included a health post, primary health centre and community health centre. Secondary health facilities include hospitals with inpatient wards, that is, district hospitals, community hospitals and private hospitals. Tertiary Hospitals are referral centres with specialist services, that is, zonal hospitals, regional hospitals, province hospitals, central hospitals, and medical colleges. Private practice includes private clinics, polyclinics with no inpatient care.

### 2.3 Sample selection

Our inclusion population included medical physicians. Medical physicians are medical officers who have completed their primary medical education, Bachelor of Medicine, and Bachelor of Surgery (MBBS) and registered in the Nepal Medical Council (NMC) with no specialist training. They are often the first point of contact for seeing COPD patients in primary, secondary and tertiary health facilities and even in private practice. Due to the vast geographical diversity in certain areas of Nepal, they are the only available doctors.

### 2.4 Sample size

The minimum sample size required was 152 and was calculated using expected proportions of 10%, an accepted error of 5%, and a nonresponse rate of 10% at a 95% confidence interval (C.I).

### 2.5 Tools

The knowledge questionnaire was designed based on the COPD GOLD 2020 guideline; however, we did literature reviews to identify background variables and intermediate variables of knowledge and practice of COPD management among physicians. The questionnaire was further discussed with the content expert from Tribhuvan University Teaching Hospital (TUTH), G. P. Koirala National Centre for Respiratory Disease, ensuring face and content validity. After pretesting it on 20 medical physicians not included in the study, we prepared the final questionnaire. It consists of demographic and work‐related characteristics; age, gender, medical qualification, employment duration, province, number of COPD cases seen per week, availability of spirometry, availability of pulmonary rehabilitation and vaccination, and participation in COPD continuing medical education (CME)/training participation. We used single and multiple‐choice questions to test the knowledge level on five domains. They are COPD epidemiology and disease definition, risk factors, COPD diagnosis, treatment and knowledge on acute exacerbations with 20 total points. Each correct response based on GOLD guidelines was given one score, and the incorrect answer was allocated zero.

To test practice, participants were asked questions on when to suspect COPD as a differential, do they do smoking screening, what is the first‐line management done for suspected COPD cases, when do they refer COPD patients for spirometry, what antibiotics are used as first‐line antibiotics for acute exacerbation of COPD, do they ask for drug adherence, what are the first‐line bronchodilators used for acute relief of symptoms and do they explain COPD action plan. Each appropriate response was given one score out of 8. The last section included the confidence level of the participants in COPD diagnosis and treatment, followed by factors causing difficulties in patients to quit smoking, proper diagnosis, and providing appropriate pharmacological therapy to COPD patients.

### 2.6 Data collection and analysis

We noted the health facilities available in Gandaki and Bagmati province from the data of the Government of Nepal, Ministry of Health and Population, Health Emergency and Disaster Management Unit, Health Emergency Operation Centre in coordination with provincial health report. From that list of health institutes, medical physicians were contacted for participation through telephone, social media, and email. Once they agreed, we sent an online self‐administered structured questionnaire in the Google form. Data were collected from May 10th 2021, to June 10th 2021. Ethical approval for this study was obtained from Nepal Health Research Council (NHRC).

We conducted a descriptive analysis with percentage, mean, median, and proportion. Knowledge and practice total scores were graded according to Blooms original cut‐off points; good (≥ 80%), moderate (60%–79%) and poor (<60%).^14^ Data were analysed with IBM SPSS statistics version 26 and Stata version 13. A correlation test was done to see the association between knowledge scores and practice scores. *p* value <0.05 were considered significant.

## 3 RESULTS

A total of 152 medical physicians participated in the study. The baseline demographic and work‐related characteristics are shown in (Table 1). Of the total participants, 73.0% were male. Most of the participants, 93.4%, belonged to the 20–30 age group. For the province, an almost equal number of participants were from Bagmati province and Gandaki province. Furthermore, 44.0% of physicians worked in primary level health facilities, followed by 23.6% from tertiary level health facilities, 18.4% from secondary health facilities and 13.8% from private practice. Additionally, 44.0% of the participants had an employment duration of less than a year, 46.0% worked for 1–2 years, and 9.6% worked for more than two years. Regarding the number of cases seen, 43.7% of the participating physicians see on average 0–5 COPD cases per week, 24.3% see 6–10 cases per week, and 30.9% see more than 10 cases in a week. It was observed that 28.9% of physicians had availability of spirometry facility within their health facility, and 40.1% had either pulmonary rehabilitation, immunisation or both services available in their workplace. Lastly, only 11.1% of physicians had received CME/training on COPD and its management during their practice.

**TABLE 1:** Baseline demographic and work‐related characteristics

Demographic variables		Frequency	Percentage
Gender	Male	111	73.0
	Female	41	26.9
Age group	20–30 years	142	93.4
	30–40 years	8	5.2
	>40 years	2	1.3
Province	Bagmati	78	51.3
	Gandaki	74	48.6
Types of health facility	Primary Level health facilities	67	44.0
	Secondary Level health facilities	28	18.4
	Tertiary Level health facilities	36	23.6
	Private Practice	21	13.8
Employment duration	<1 year	67	44.0
	1–2 years	70	46.0
	>2 years	15	9.6
No. of COPD cases seen per week	0–5 cases per week	68	43.7
	6–10 cases per week	37	24.3
	>10 cases per week	47	30.9
Availability of spirometry	Yes	44	28.9
	No	108	71.0
Availability of pulmonary rehabilitation and Immunisation	Either one or both	61	40.1
	None	91	56.8
Have received CME or training in COPD	Yes	17	11.1
	No	135	88.8

Abbreviations: CME, continuing medical education; COPD, chronic obstructive pulmonary disease.

### 3.1 Knowledge analysis

Out of a total score of twenty, the mean score for overall COPD knowledge was 17.8 ± 2.4. Using Bloom's original cut‐off point for knowledge grading, 45.4% of physicians have good knowledge, 47.3% have moderate, and 7.2% of the medical physicians have poor knowledge regarding COPD. (Table 2) Shows the average mean score of overall knowledge and each domain of knowledge tested. 90.7% of the physicians correctly chose that the burden of COPD is increasing globally. Similarly, 65.7% said the burden of COPD in Nepal is within the top five causes of morbidity. For disease definition, most of the participants, 88.8%, correctly defined COPD as a chronic preventable and treatable condition. Only 32.9% of the participants correctly identified all six risk factors for COPD: smoking, exposure to biomass fuel, exposure to outdoor pollution, occupational air pollutions, genetic factors like alpha 1 anti‐trypsin deficiency and recurrent chest infections. Smoking was the most selected risk factor by 98.0%, whereas recurrent chest infection was the least selected by 48.6%.

**TABLE 2:** Participants overall knowledge score and on each domain of study

Knowledge on	Maximum available score	Mean score	*SD*
Overall COPD disease and management	20	17.8	2.4
COPD epidemiology and definition	3	2.7	0.6
COPD risk factors	6	4.9	1.2
COPD diagnosis	2	1.0	0.5
COPD treatment	5	3.2	0.8
Acute exacerbation of COPD	4	3.1	0.9

Abbreviation: COPD, chronic obstructive pulmonary disease.

Regarding the gold standard test for COPD diagnosis, 86.1% of the participants chose spirometry rightly; however, only 21.0% of the participants were able to identify the correct spirometry cut‐off value for diagnosis, that is, post‐bronchodilator FEV1/FVC < 0.7. For the use of inhaled corticosteroids (ICSs), 86.8% of physicians knew the history of two or more episodes of acute exacerbation in a year, and 6.5% of the physicians knew eosinophils count >300 cells/μl an indication for its use. The majority of the participants, 61.8%, correctly identified that oxygen supplementation and smoking cessation help decrease mortality in COPD patients. Regarding the use of domiciliary oxygen therapy, 74.3% of physicians rightly said arterial hypoxemia with paO_2_ < 55, spO_2_ < 88% as an indication for its use. Of the participants, 41.4% chose all four given symptoms of acute exacerbation, that is, increasing shortness of breath, increasing cough, increasing mucus production and mucus colour change. The most selected symptoms were increasing shortness of breath, 92.7%, and increasing cough, 89.4%.

### 3.2 Practice analysis

For practice, out of possible eight, the mean score of the participants was 5.30 ± 1.30. Using Bloom's original cut‐off point for practice grading, 30 (19.73%) of the respondents were categorised as having a good practice, 55.9% at the moderate level, and 24.4 with poor practice in diagnosing and managing COPD. (Table 3) shows the overall response of the participants in different practice questions. Of respondents, 75.6% think of COPD as a differential when a patient with cough and difficulty breathing presents to the clinic. Similarly, 80.2% of the physicians said they enquired about smoking history in every patient age greater than 20 years.

**TABLE 3:** Frequency distribution of participants practice in COPD

A. Do you think of COPD as differentials when a patient age greater than 35 years presents with a history of cough and difficulty in breathing?	
1. Yes	115 (75.6%)
2. No	37 (24.3%)
B. Do you ask about smoking history in every patient aged greater than 20 years?	
1. Yes	122 (80.2%)
2. No	30 (19.7%)
C. What management approach would you do in your practice for a suspected COPD patient with a history of difficulty in breathing, cough and smoking?	
1. Perform PFT if available or refer the patient to a spirometry facility	56(36.8%)
2. Give a trial of short/long‐acting bronchodilator and send home	7 (4.6%)
3. Do X‐ray, blood test and give a trial of bronchodilator and send home	89 (58.5%)
D. When do you refer the diagnosed COPD patient for spirometry?	
1. Whenever the patient is not improving with the medical therapy	97 (63.8%)
2. Not at all	55 (36.1%)
F. Do you ask about the drug adherence of COPD patients during follow up?	
1. Yes	141 (92.7%)
2. No	11 (7.2%)
G. What do you use as a first‐line medication for acute relief of shortness of breath in COPD patients?	
1. SAMA alone or combination with SABA	91(59.8%)
2. LAMA alone or combination with LABA	52 (34.2%)
3. Inhaled corticosteroids alone or in combination LAMA/LABA	36 (23.6%)
E. What first‐line antibiotics do you use in AECOPD?	
1. Amoxicillin or azithromycin	133 (87.5%)
2. Levofloxacin	16 (10.5%)
3. Cefixime	3 (1.9%)
H. Do you counsel the COPD patient of the COPD action plan at home?	
1. Yes	57 (37.5%)
2. No	95 (62.5%)

Abbreviation: COPD, chronic obstructive pulmonary disease.

For initial management of suspected COPD, 58.5% said they would perform an X‐ray, blood test and give a trial of bronchodilator and send them home, whereas 36.8% of the participants chose to perform pulmonary function test (PFT) or refer patients to spirometry facility. Furthermore, 63.8% said that not improving to medical therapy is the main reason for referring COPD patients to spirometry. The first‐line drug of choice to treat acute relief of symptoms, 59.8% of physicians, chose to use short‐acting muscarinic antagonist (SAMA) alone or in combination with short‐acting beta 2 agonists (SABA) to relieve shortness of breath whereas, 23.6% of the physician choose to use ICS alone or in combination. Similarly, 87.5% used either amoxicillin or azithromycin as a first‐line antibiotic for AECOPD. During follow‐up, 92.7% of the participants asked about medical adherence in COPD patients; however, only 37.5% said they would counsel them about the COPD action plan at home during an acute exacerbation.

### 3.3 Correlation analysis

We did Pearson correlation, and there was a statically significant very low positive correlation between total knowledge score and practice score with *r* = 0.18 and *p* value <0.02.

### 3.4 Factors affecting difficulties in patients quitting smoking

Table 4 shows the reasons that are preventing the physician from helping COPD patients quit smoking. The most common factors were lack of follow‐up 65.7%, and patients not wanting to discuss a quit plan 65.1%. Others included lack of access to pharmacological measures like nicotine replacement therapy (NRT) 37.5% and physicians not being aware of any quit plan 8.5%.

**TABLE 4:** Factors affecting difficulty in patients quit smoking

Factors	Frequency (%)
1. Lack of patient follows up.	100 (65.7%)
2. Patients do not want to discuss a quit plan.	99 (65.1%)
3. Lack of asses to pharmacological measures like Nicotine replacement therapy (NRT).	57 (37.5%)
4. Physician not aware of any quit plan.	13 (8.5%)
5. Physician not aware that quit plan needs to be addressed.	2 (1.3%)

### 3.5 Confidence level in diagnosis and factors preventing a proper diagnosis of COPD

Of the doctors, 74.9% were either confident or extremely confident about correctly diagnosing the COPD cases. Table 5 shows the factors preventing a proper diagnosis of COPD. Most physicians said that lack of patient follow‐up 71.7%, lack of screening device for COPD 65.7%, and lack of professional training in the diagnosis of COPD 61.1% are the main reasons. Other reasons included lack of spirometry nearby in the referral centres 58.5% and poor patients' financial status 53.2%.

**TABLE 5:** Factors preventing the proper diagnosis of chronic obstructive pulmonary disease (COPD) patients

Factors	Frequency (%)
1. Lack of patient follow up.	109 (71.7%)
2. Lack of screening devices in the health facility.	100 (65.7%)
3. Lack of professional training in COPD diagnosis.	93 (61.1%)
4. Lack of spirometry nearby in a referral centre.	89 (58.5%)
5. Poor financial status of the patients.	81 (53.2%)
6. Lack of screening questions.	73 (48.0%)
7. Patient self‐medicate from the pharmacy.	52 (34.2%)
8. Lack of time in the practice for detailed patient evaluation.	51 (33.5%)
9. Shortage of appropriate medication.	31 (20.3%)

### 3.6 Confidence levels in pharmacological treatment and factors affected in providing appropriate pharmacological therapy to COPD patients

Almost 83.6% of the physicians said that they were either undertreating, unsure or overtreating COPD patients. Table 6 shows the factors influencing in providing appropriate treatment to COPD patients. The most chosen factors were lack of professional training in COPD disease management, poor follow‐up and high medication cost.

**TABLE 6:** Factors affecting appropriate pharmacological therapy to chronic obstructive pulmonary disease (COPD) patients

Factors	Frequency (%)
1. Lack of professional training in COPD disease management.	59 (38.8%)
2. Poor follow up by the patients.	43 (28.2%)
3. Cost of medication and poor financial status of the patients.	24 (15.7%)
4. Lack of proper access to the prescribed medication by patients.	20 (13.1%)
5. Patient self‐medicate	6 (3.9%)

## 4 DISCUSSION

Our study showed that the overall knowledge of COPD based on GOLD guidelines among medical physicians is good in Nepal's Bagmati and Gandaki provinces. Still, the practice level was not up to the knowledge they had. There was a significant, very low positive correlation between total knowledge and practice scores. These can be due to various patient‐related factors like a poor follow‐up, health professional‐related factors like lack of training in COPD, and health institute factors like unavailability of screening devices, spirometry, and medications. They all can act as a barrier to diagnosing and managing COPD properly. These factors were in line with a report from the American Thoracic Society on challenges in implementing COPD guidelines in LMIC.^15^ Thus, overcoming those barriers by proper training and supply of resources in LMIC like Nepal can help increase the physician's practice level in COPD and reflect their knowledge.

There are no previous studies available in COPD knowledge among medical physicians from Nepal; however, the level of knowledge was higher than the study from Saudi Arabia that showed physicians had a fair understanding of COPD based on GOLD guidelines.^16^ In our study, good physicians' knowledge was there even though many participants had not received CME or training on COPD. Most of the participants were practising for less than 2 years immediately after graduation from medical college as per requirement from Nepal Government. Earlier years of practice after graduation from the medical college might have influenced their overall knowledge of COPD. The knowledge acquired from a medical college can decline over time, so it is crucial to provide continuous training to medical physicians to enhance their expertise. Similarly, in subdomains of knowledge, most physicians had good knowledge in each domain except for diagnosis. More than two‐thirds of the medical physicians were aware of smoking and exposure to biomass fuel as risk factors for COPD. These two are the most common risk factors of COPD prevalent in South‐East Asia.^6^ Knowledge of these risk factors will help them select a high‐risk population. Similarly, they were aware of the indications for ICS use in COPD despite different studies suggesting its inappropriate use by physicians to treat COPD.^17^, ^18^ In addition, the two most selected interventions for treatment to reduce mortality in COPD patients, were smoking and domiciliary oxygen therapy.

For knowledge in the diagnosis, many physicians were aware of spirometry as the gold standard test for COPD diagnosis. Still, only a few could correctly choose its cut‐off value. It can be due to many physicians were practising in settings with no spirometry, that is, primary and secondary health facilities. Lack of spirometer, poor or no teaching of spirometry reading in the medical school, and lack of evidence base demonstration of the value might have resulted in less knowledge on spirometry correlation.^19^ Proper hands‐on training and exposure to spirometry can help them correlate the cut‐off value for the diagnosis.

We further studied the practice and confidence level of physicians in the diagnosis and treatment of COPD. Although having good knowledge of COPD based on GOLD guidelines, the practice was not within guideline recommendations, especially in spirometry use. This finding was similar to a previous study from Nigeria that showed despite having an adequate understanding of the GOLD COPD guideline among physicians, adherence to the guideline recommendation was very poor.^20^ Similarly, a study on primary care physician perception on the diagnosis and management of COPD in diverse regions of the world showed that management of COPD was well below guideline‐recommended levels in most of the areas investigated.^21^ Only a few participants considered doing spirometry to diagnose COPD. They would use a trial of bronchodilators instead of spirometry for suspected COPD cases, and the majority of the participants were confident in their diagnostic approach. It can be due to the unavailability of the spirometry facility. Most of the physicians in our study did not have the availability of spirometry or peak flow spirometry within the facility. Lack of spirometry facilities and no education on its use may influence how they diagnose COPD in practice and not stick to the guideline.^3^, ^22^ Furthermore, with Nepal's huge geographical diversity, the financial cost of being referred to the centre can be high compared with just the treatment; therefore, physicians and patients may be less reluctant for its use. Further study is needed to look at the practice trend after making spirometry facilities readily available to a medical physician.

However, in this current scenario for poor‐resource settings like Nepal, recommendations from Hurst et al. can be adopted to diagnose COPD. As recommended, the concerned authority and government should develop evidence‐based diagnostic tools, such as screening questionnaires and mobilise locally available resources like peak flow meters (PFMs) to diagnose COPD.^15^ These methods can be cost‐effective and readily available in LMIC when there is limited access to spirometry. Training should still be given to medical physicians in the proper use of PFM. Furthermore, the government of Nepal has a Package of Essential Non‐Communicable Disease (PEN) for early detection of chronic diseases in primary level health facilities.^23^ Hence, based on the above recommendation, PFM can be added to PEN to identify COPD patients early. These recommendations, as mentioned above, can help because many participants in our study thought of COPD as a differential when a patient with cough, shortness of breath, and smoking history presents but lacks proper diagnosis methodology. Thus, they can use the questionnaire and PFM on high‐risk patients to provisionally diagnose COPD and start proper management in case of a referral to spirometry acts as a barrier in appropriately diagnosing and managing COPD patients.

Next, even though many of the participants mentioned the correct indication when to add ICS in therapy and said SAMA/SABA to be used for acute relief of symptoms, many were unsure about their treatment approach. This insecurity in treatment could be due to a lack of training or CME on COPD and less familiarity with the different medical treatments available. Furthermore, this low level of confidence in COPD treatment can decrease the quality of community between patients and physicians. Decreased quality of conversation can lead to poor understanding of treatment from the patients regarding reasons, the timing of use, and the dose of a particular given medication.^24^ Apart from that, the cost of medicines and poor patient financial status can lead to poor compliance with medication. Thus, as recommended by Hurst et al., provision of COPD medication in essential drug list and continuous supply of it to primary and secondary level health facilities can help address those barriers.^15^ And to engage patients in self‐management programmes for COPD, the physician should be given proper hands‐on training on the drugs available and their mode of use.

Another interesting finding was that although most physicians are aware of smoking as a risk factor and ask about it in daily practice, lack of patient follow‐up and patients' unwillingness to quit was responsible for the difficulties in helping patients quit smoking. This finding was consistent with a multinational qualitative study on why physicians lack engagement with smoking cessation treatment in COPD patients that highlighted unwillingness to quit and poor follow‐up of the patients as the primary reasons.^25^ Poor follow‐up of patients can be due to socio‐economic factors, health system‐related, condition‐related, therapy‐related or patient‐related factors.^26^ Community‐level awareness about COPD, risk factors, and easy availability of treatment like NRT can help the patient come to health care attention and address smoking and COPD accordingly.

At last, a COPD action plan at home is one of the critical steps in managing COPD, but only one‐third of the participant physicians were aware of it and discussed it with their patients. Studies have shown that COPD action plans help people with COPD recognise and initiate appropriate treatment at home.^27^ Early intervention during acute exacerbation improves morbidity and mortality.^28^ Therefore, physicians should also be trained in preparing action plans for COPD patients.

Overall, early detection of airflow limitation and treatment helps reduce the burden of COPD and improve patients' quality of life.^29^ Physicians who participate in CME programmes on COPD diagnosis, staging and treatment are more likely than nonparticipants to deliver evidence‐based COPD management.^30^ Hence, in the first place for LMIC like Nepal, concerned authorities should give physicians regular training based on the availability of local resources. Second, proper diagnostic infrastructure should be in place to improve the early diagnosis of COPD cases. Third, COPD medication should be on the essential drug list and regularly supplied to primary and secondary level hospitals. Apart from that, adequate disease awareness is also required in patients to increase their follow‐up and make them aware of the harmful effects of smoking.

## CONFLICT OF INTEREST

The authors declare there is no conflict of interest present.

## FUNDING INFORMATION

No funding was received for this research.

## ETHICS STATEMENT

Ethical approval for this study was provided by the Government of Nepal, Nepal Health Research Council (Proposal ID: 636‐2020). Informed consent to participate and publication was obtained from all individual participants included in the study.

## AUTHOR CONTRIBUTIONS

Suraj Ghimire, Anish Lamichhane, Anita Basnet, Samikshya Pandey and Ram Kumar Shrestha designed the study and questionnaire. Nahakul Poudel, Bushan Shrestha, Santosh Pathak and Gaurav Mahato did data collection and entry. Suraj Ghimire and Anita Basnet were involved in data analysis and manuscript writing.

## Data Availability

The data that support the findings of this study are available from the corresponding author upon reasonable request.
